# Expiratory flow limitation development index (ELDI): a novel method of assessing respiratory mechanics in COPD

**DOI:** 10.1186/s12931-024-02972-2

**Published:** 2024-10-03

**Authors:** James Dean, Stephen J. Fowler, Dave Singh, Augusta Beech

**Affiliations:** 1 Division of Infection, Immunity and Respiratory Medicine, School of Biological Sciences, Faculty of Biology, Medicine and Health, Manchester Academic Health Science Centre, The University of Manchester, Manchester University NHS Foundation Trust, Manchester, M23 9LT UK; 2 Medicines Evaluation Unit, Southmoor Road, Manchester, M23 9QZ UK

**Keywords:** COPD, Oscillometry, Expiratory flow limitation, Respiratory mechanics, Airway remodelling

## Abstract

**Background:**

Expiratory flow limitation (EFL) can be detected using oscillometric reactance and is associated with a worse clinical presentation in chronic obstructive pulmonary disease (COPD). Reactance can show negative swings upon exhalation, which may develop at different rates between patients. We propose a new method to quantify the rate of EFL development; the EFL Development Index (ELDI).

**Methods:**

A retrospective analysis of data from 124 COPD patients was performed. Data included lung function tests, Impulse Oscillometry (IOS), St Georges Respiratory Questionnaire (SGRQ), modified Medical Research Council (mMRC) scale and COPD Assessment Test (CAT) score. Fifty four patients had repeat data after 6 months. Twenty two patients had data recorded after 5 days of treatment with long acting bronchodilator therapy. EDLI was calculated as the mean expiratory reactance divided by the minimum expiratory reactance.

**Results:**

The mean ELDI was used to categorise patients with rapid onset of EFL (> 0.63; n = 29) or gradual onset (≤ 0.63; n = 34). Those with rapid development had worse airflow obstruction, lower quality of life scores, and greater resting hyperinflation, compared to those with gradual development. In patients with EFL, ELDI correlated with symptoms scores, airflow obstruction, lung volumes and gas diffusion. Both EFL and ELDI were stable over 6 months. EFL and EDLI improved with bronchodilator treatment.

**Conclusions:**

COPD patients with rapid EFL development (determined by ELDI) had worse clinical characteristics than those with gradual EFL development. The rate of EFL development appears to be associated with clinical and physiological characteristics.

**Supplementary Information:**

The online version contains supplementary material available at 10.1186/s12931-024-02972-2.

## Take home message

We propose a new method of quantifying the rate of EFL development (EFL Development Index; ELDI), and report that those with rapid EFL development upon exhalation have worse physiological and clinical characteristics.

**Supplementary Information:**

The online version contains supplementary material available at 10.1186/s12931-024-02972-2.

## Introduction

COPD is a heterogeneous condition characterised by chronic respiratory symptoms and airflow obstruction arising from abnormalities of the airways and/or alveoli [1]. Widespread pathological changes are observed in the peripheral small airways, such as airway wall thickening, mucus plugging and airway collapse due to the loss of supporting alveolar attachments [2, 3].

Respiratory oscillometry measures the mechanical properties of the respiratory system providing information on resistance and reactance, with the latter representing stiffness of the lung periphery [4]. Small airway collapse during expiration can cause expiratory flow limitation (EFL) [5]. This has been quantified using reactance measurements, with the difference between mean inspiratory reactance (X5_in,mean_) and mean expiratory reactance (X5_ex,mean_) ≥ 0.28 kPa.s.L^−1^ (∆X5) identifying COPD patients with EFL [6]. The presence of EFL is associated with greater hyperinflation and gas trapping, and a higher impact on daily living [6–8].

Previous studies have commonly assessed the magnitude of EFL using ∆X5, although the maximum shift in reactance between inspiration and expiration (X5_peak-peak_) is an alternative method, using a threshold value of ≥ 0.59 kPa.s.L^−1^ [6]. The magnitude of EFL has been the focus of recent studies; less is known about the rate of EFL development. It has been proposed that rate of EFL development upon exhalation can differ between patients with COPD [9, 10], but this has not been quantified.

**Fig. 1:**
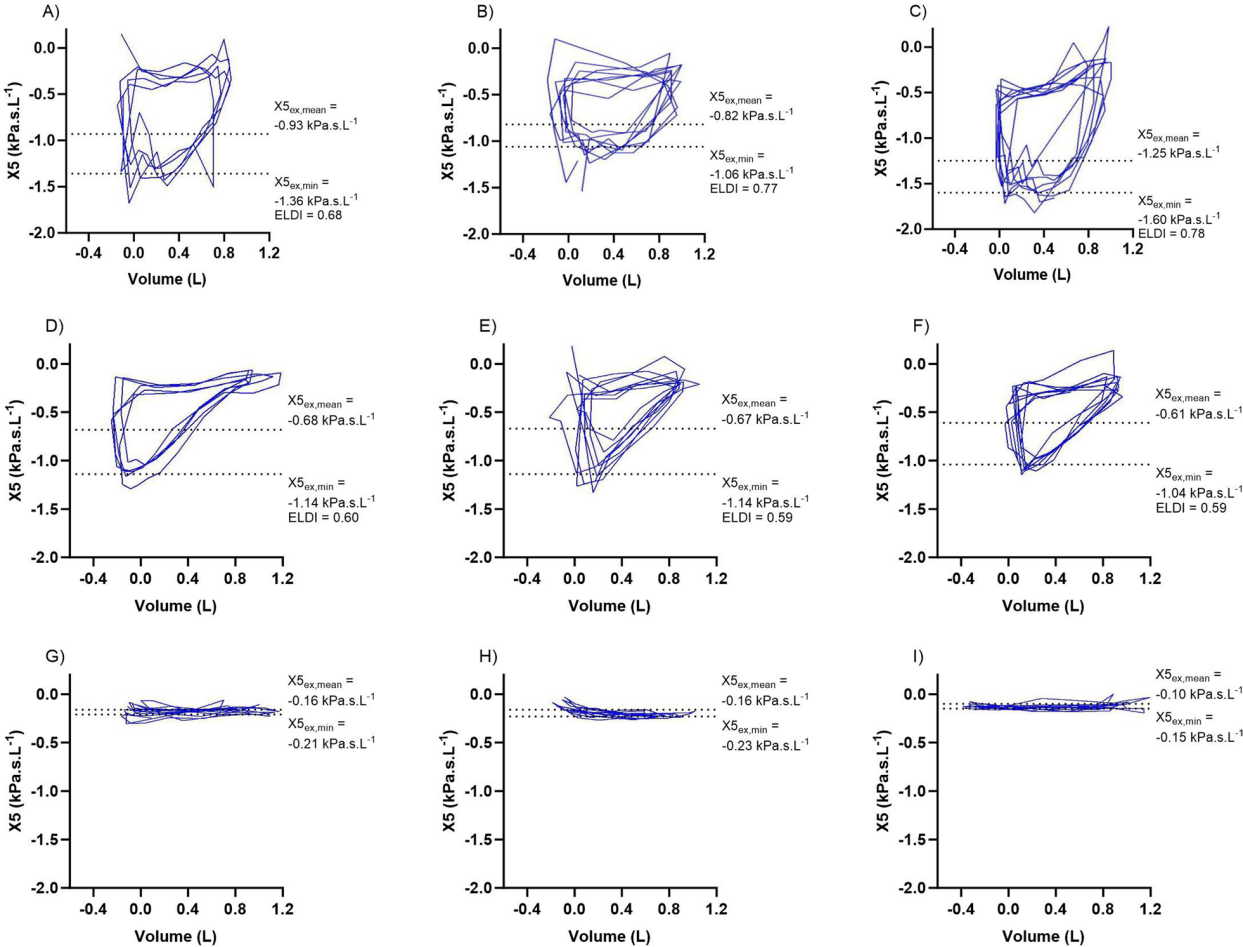
Reactance-volume loops during tidal breathing for 9 COPD patients from the data set, showing rapid EFL (**A**–**C**), gradual EFL (**D**–**F**), and no EFL (**G**–**I**). During inhalation volume increase (moves right), and during exhalation volume decreases (moves left). ELDI calculated as X5_ex,mean_/X5_ex,min_. ELDI was not calculated for subjects with no EFL. EFL reported as ∆X5 (mean difference ≥ 0.28 between inspiration and expiration X5) and X5_peak-peak_ (maximum difference ≥ 0.59 between inspiration and expiration X5). Parameters are reported as the average of multiple breaths. For example, X5_ex,min_ is not aligned with the lowest point on each of the graphs, as it includes the minimum X5 during previous and subsequent exhalations. Note. images exported from IOS device, imported into Prism software and aligned with correctly to x and y axes. X5: reactance at 5 Hertz; X5_ex,mean_: mean X5 during expiration; X5_ex,min_: minimum X5 during expiration; ELDI: EFL Development Index

Heterogeneity in EFL development can be observed by inspection of the reactance-volume loop derived from respiratory oscillometry (Fig. 1). A rapid onset of EFL (EFL^rapid^) with a drop in reactance close to the onset of exhalation produces a more rectangular loop (Fig. 1A–C). In contrast, a gradual onset of EFL (EFL^gradual^) with a drop in reactance later in exhalation produces a more triangular loop (Fig. 1D–F). No EFL is indicated by a flat loop where reactance is similar during inhalation and exhalation (Fig. 1G–I). We propose that a calculation of the ratio of mean expiratory reactance to the peak negative expiratory reactance _(_X5_ex,mean_/X5_ex,min_; Fig. 1; Figure S1) can provide information regarding the loop shape, with higher values closer to 1 signifying rapid EFL onset. We describe this as the EFL Development Index (ELDI).

We have investigated the potential clinical utility of the ELDI in COPD patients. We report differences in clinical characteristics according to ELDI, the stability of ELDI over time and whether ELDI changes with inhaled treatment.

## Study design and methods

**Fig. 2:**
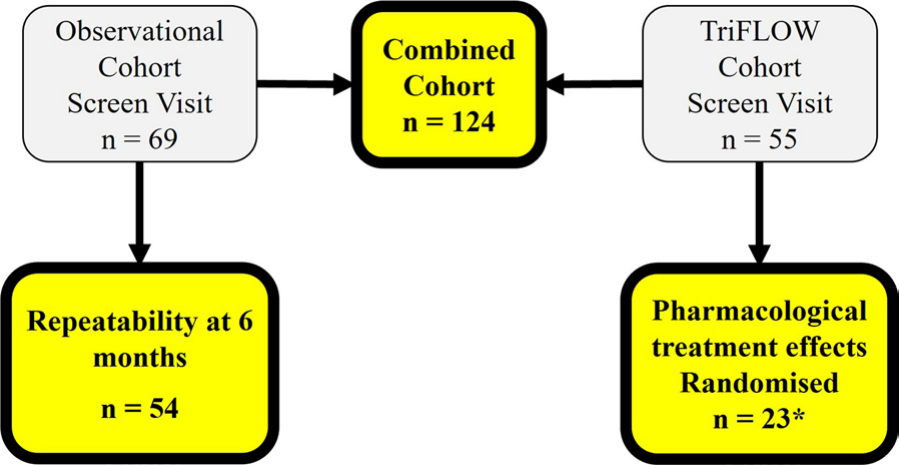
Flow diagram of COPD patients selected for analysis. The combined cohort comprised of screening data from both the TriFlow study and Observational study, of patients with acceptable oscillometry and spirometry measurements. Further details of the randomised clinical trial design can be found in the supplement. *n = 1 withdrawn due to poor treatment compliance

We performed a retrospective analysis of oscillometry data collected from two previous studies at the Medicines Evaluation Unit (Manchester University NHS Foundation Trust, Manchester, UK); an observational cohort of highly symptomatic COPD patients with repeated measurements [8] and the TriFLOW clinical trial of inhaled triple therapy [11]. Screening data from both studies was used for a combined analysis (Fig. 2). Data from the observational cohort was used to assess repeatability after 6 months. Data from patients randomised to the TriFLOW study was used to assess treatment response (Fig. 2, further detail in figure S2). All patients provided written informed consent using protocols approved by local Ethics Committees (16/NW/0836; 18/NI/0194). Patients met the global initiative for chronic obstructive lung disease (GOLD) criteria for the diagnosis of COPD [1], were aged ≥ 40 years old, had a smoking history of ≥ 10 pack years, and a forced expiratory volume in 1 s (FEV_1_) / forced vital capacity (FVC) ratio < 0.7. Patients with a history of asthma were excluded. Further details for each cohort are available in the supplemental material.

The following assessments were performed, in the following order; symptom questionnaires (see supplement), impulse oscillometry (IOS), pre bronchodilator spirometry, body plethysmography, diffusing capacity of the lungs for carbon monoxide (DLCO, observational cohort only), post bronchodilator spirometry. Procedures were repeated at 6 months in the observational cohort. In the TriFLOW cohort, procedures were repeated after 5 days of treatment with either Beclometasone Dipropionate/Formoterol (BDP/F) or Beclometasone Dipropionate/Formoterol/Glycopyrronium (BDP/F/G), in a randomised crossover design with a washout period between treatments (BDP and short-acting β2 agonist only). At baseline (BDP only) and on day 5 of each treatment, lung function measurements were performed for 12 h following the final morning dose. Full details are available in the supplement.

Spirometry (Easy On-PC spirometer, NDD medical technologies, Zurich, CHE), body plethysmography and DLCO (both Vmax, CareFusion, Höchberg, DEK) were performed according to American Thoracic Society (ATS)/European Respiratory Society (ERS) guidelines [12–15]. IOS (MasterScreen; Erich Jaeger, Höchberg, DEK) was performed as previously described [16], to ERS guidelines [4, 17]. See supplement for full details of procedures. EFL was calculated using within breath analysis of reactance data, defined as a ΔX5 ≥ 0.28 kPa.s.L^−1^ or as a X5_peak-peak_ ≥ 0.59 kPa.s.L^−1^. The latter was used in the current study to allow the calculation of speed of EFL development, which is made in relation to the peak EFL during exhalation (i.e. X5_ex,min_). ELDI was calculated as the mean expiratory reactance (X5_ex,mean_), divided by the minimum expiratory reactance (X5_ex,min_), averaged over all breaths (X5_ex,mean/min_). Figure S1 shows the derivation of ∆X5, X5_peak-peak_ and ELDI.

In those individuals with EFL, two groups were identified using the mean ELDI value as a cutoff: EFL^rapid^ with rapid development (ELDI > mean) and EFL^gradual^ with gradual development (ELDI ≤ mean). Data distributions were determined by the D’Agostino and Pearson normality test. Comparisons between the EFL^rapid^, EFL^gradual^ and no EFL (EFL^no^) groups were assessed using one-way ANOVA with Tukey’s multiple comparisons test, or Kruskal–Wallis test with Dunn’s multiple comparisons test, as appropriate. Correlations between ELDI and EFL measures were assessed with Pearson correlation coefficient. Repeatability was assessed using paired two-way t-test, Wilcoxon singed rank test, Pearson correlation coefficient, Spearman correlation coefficient, and intraclass correlation coefficient (ICC) analysis were adjusted using Log(X + 1) and interpreted as previously described [18]. Treatment effect was assessed using repeated measures one-way ANOVA with Tukey’s multiple comparisons test. Data distribution, comparison analysis, and correlation analysis was performed using Prism 9 (Graphpad, LA Jolla, CA, USA). ICCs were based on an absolute agreement, two-way mixed effects model, using SPSS 25 (IBM, Armonk, NY, USA). Significance was determined as p < 0.05.

## Results

Data from 124 COPD patients were available for analysis; 69 from an observational cohort and 55 from the TriFLOW study, of which 22 patients were randomised (Fig. 2).

### Baseline characteristics

Baseline characteristics of the overall cohort are shown in Table 1. Amongst individuals with EFL, the mean ELDI was 0.63 (range: 0.38–0.80), which was used to categorise patients as EFL^rapid^ (> 0.63; n = 29) or EFL^gradual^ (≤ 0.63; n = 34). Examples of the shape of the reactance-volume loops within these ELDI categories are shown in Fig. 1.

**Table 1:** Baseline characteristics of the combined cohort

	EFL^rapid^ (n = 29)	EFL^gradual^ (n = 34)	p	EFL^no^ (n = 61)	p vs EFL^rapid^	p vs EFL^gradual^
Age (years)	66 [59–71]	69 [65–72]	0.34	65 [60–71]	> 0.99	0.32
Gender (% male)	52	56	> 0.99	59	> 0.99	> 0.99
BMI (kg/m^2^)	27.4 (5.8)	29.3 (5.0)	0.28	26.7 (4.2)	0.81	0.0390
Pack yrs	39.1 [29.9–54.4]	45.3 [34.5–54.5]	> 0.99	36.6 [28.8–47.8]	0.89	0.12
Prescribed LABA (%)	83	91	0.93	89	> 0.99	> 0.99
Prescribed LAMA (%)	93	85	> 0.99	79	0.25	> 0.99
Prescribed ICS (%)	86	88	> 0.99	84	> 0.99	> 0.99
Exacerbations (0/12)	0 [0–1]	1 [0–1]	> 0.99	1 [0–1]	0.97	> 0.99
Pre-BD FEV_1_%pred	42.5 (12.1)	54.1 (19.6)	0.0253	63.3 (15.6)	< 0.0001	0.0443
Pre-BD FEV_1_ Z score	− 3.68 (0.80)	− 2.89 (1.03)	0.0055	− 2.49 (0.92)	< 0.0001	0.17
Pre-BD FVC %pred	87.1 (10.0)	86.8 (19.9)	> 0.99	93.3 (15.0)	0.24	0.19
Pre-BD FVC Z score	− 1.35 (0.66)	− 1.29 (1.12)	0.97	− 0.83 (0.95)	0.06	0.10
Pre-BD FEV_1_/FVC (%)	36.2 [30.8–47.9]	49.5 [39.6–60.1]	0.0106	54.9 [46.7–62.0]	< 0.0001	0.29
Pre-BD FEV_1_/FVC Z score	− 4.29 [− 4.82–− 3.76]	− 3.45 [− 3.96–− 2.18]	0.0055	− 2.75 [− 3.44–− 2.11]	< 0.0001	0.48
Pre-BD FEF_25%-75%_ %pred	11.5 [8.0–16.3]	18.0 [10.5–29.5]	0.07	24.0 [16.8–35.3]	< 0.0001	0.10
Pre-BD FEF_25%-75%_ Z score	− 3.57 [− 3.95–− 3.14]	− 3.05 [− 3.48–− 2.29]	0.0222	− 2.71 [− 3.09–− 2.06]	< 0.0001	0.53
CAT score	21.8 (7.4)	21.3 (6.7)	0.97	19.7 (6.7)	0.42	0.56
mMRC	3 [1–4]	4 [2–4]	0.66	3 [1–4]	0.63	0.14
SGRQ symptoms	69.0 (18.5)	66.6 (13.0)	0.88	66.0 (17.0)	0.81	0.99
SGRQ activity	80.9 (16.0)	72.5 (18.9)	0.28	65.3 (16.2)	0.0093	0.30
SGRQ impact	51.2 (10.6)	38.2 (19.7)	0.0405	32.2 (16.8)	0.0008	0.41
SGRQ total	63.5 (9.5)	53.6 (16.0)	0.07	48.4 (14.6)	0.0017	0.38
TLC %pred	111.9 (15.4)	105.1 (16.5)	0.15	101.2 (11.5)	0.0032	0.41
TLC Z score	0.86 (1.21)	0.37 (1.25)	0.16	0.08 (0.91)	0.0057	0.23
FRC %pred	145.2 [125.7–170.8]	110.3 [98.6–154.9]	0.0120	110.8 [95.7–126.4]	< 0.0001	0.47
FRC Z score	1.72 (1.26)	0.92 (1.46)	0.0234	0.50 (1.03)	< 0.0001	0.12
RV %pred	181.0 (54.0)	151.6 (42.9)	0.0209	128.9 (34.1)	< 0.0001	0.0388
RV Z score	1.99 [1.21–3.53]	1.51 [0.41–2.92]	0.28	0.82 [− 0.06–1.78]	< 0.0001	0.05
RV/TLC %pred	161.1 (30.3)	143.7 (25.9)	0.0499	128.2 (28.6)	< 0.0001	0.0373
RV/TLC Z score	2.79 [1.73–3.87]	1.85 [1.39–3.25]	0.31	1.34 [0.20–2.40]	< 0.0001	0.05
DLCO %pred	54.1 (19.6)	61.6 (23.4)	0.57	60.2 (24.9)	0.66	0.97
DLCO Z score	− 3.34 [− 4.57–− 2.78]	− 3.13 [− 4.26–− 0.82]	0.58	− 3.47 [− 4.90–− 1.78]	> 0.99	> 0.99
KCO %pred	65.7 (25.11)	75.8 (23.0)	0.45	69.8 (28.7)	0.86	0.69
KCO Z score	− 2.85 [− 3.73–− 1.53]	− 1.74 [− 2.76–0.21]	0.23	− 2.29 [− 3.88–− 0.76]	> 0.99	0.43
VA %pred	83.1 (11.5)	79.9 (12.5)	0.68	86.8 (11.3)	0.54	0.10
VA Z score	− 1.44 (0.98)	− 1.75 (1.13)	0.44	− 1.09 (0.96)	0.44	0.08
∆X5 (kPa/L/s)	0.57 [0.34–0.78]	0.39 [0.30–0.51]	0.59	0.03 [0.00–0.08]	< 0.0001	< 0.0001
X5peak-peak (kPa/L/s)	1.02 [0.73–1.44]	1.07 [0.83–1.31]	> 0.99	0.26 [0.16–0.40]	< 0.0001	< 0.0001
R5-R20 (kPa/L/s)	0.33 (0.08)	0.29 (0.10)	0.11	0.11 (0.08)	< 0.0001	< 0.0001
AX (kPa/L)	4.36 [3.87–5.24]	3.39 [2.81–5.34]	0.47	1.21 [0.69–1.98]	< 0.0001	< 0.0001
fRES (Hz)	29.3 (3.6)	28.3 (5.6)	0.75	20.9 (6.0)	< 0.0001	< 0.0001
ELDI	0.69 [0.66–0.74]	0.58 [0.55–0.61]	< 0.0001	*n/a*	*n/a*	*n/a*

Missing data: smoking history, rapid n = 1, gradual n = 4, non n = 13; exacerbation history, rapid n = 2, gradual n = 3, non n = 13; spirometry, rapid n = 3, gradual n = 5, non n = 15; reversibility, rapid n = 3, gradual n = 5, non n = 17; CAT & mMRC, rapid n = 1, gradual n = 3, non n = 13; SGRQ, rapid n = 11, gradual n = 12, non n = 32; lung volumes, rapid n = 1, gradual n = 2, non n = 2; gas exchange, rapid n = 11, gradual n = 12, non n = 32

*AX* reactance area, *BD* bronchodilator, *BMI* body mass index, *CAT* COPD assessment test, *DLCO* diffusing capacity for carbon monoxide, *ELDI* EFL development index, *FEV1* forced expiratory volume in 1 s, *FRC* functional residual capacity, *FVC* forced vital capacity, *fRES* resonant frequency, *ICS* inhaled corticosteroids, *KCO* carbon monoxide transfer coefficient, *LABA* long acting beta agonist, *LAMA* long acting muscarinic antagonist, *mMRC* modified medical research council questionnaire, *RV* residual volume, *R5* resistance at 5 Hz, *R20* resistance at 20 Hz, *SGRQ* St George’s respiratory questionnaire, *TLC* total lung capacity, *VA* alveolar volume, *X5* reactance at 5 Hz *∆X5* difference in total reactance between inspiration and expiration

Approximately half of the cohort (n = 61) displayed no EFL (X5_peak-peak_ < 0.59 kPa.s.L^−1^). The absence of EFL in this group was confirmed by a lower mean ΔX5 (0.03 kPa.s.L^−1^), compared to the EFL^rapid^ (0.57 kPa.s.L^−1^) and EFL^gradual^ (0.39 kPa.s.L^−1^) groups (p < 0.0001 for both comparisons). The EFL^no^ group displayed less small airway resistance (estimated by R5-R20) and loss of peripheral airway distensibility (estimated by AX) compared to both EFL^rapid^ and EFL^gradual^ groups (p < 0.0001 for all comparisons). These measurements, and other oscillometry measurements (aside from ELDI) were similar in the EFL^rapid^ and EFL^gradual^ groups (Table 1). Similarly, there were no associations between ELDI and X5_peak-peak_ or ΔX5 (Figure S3).

The EFL^rapid^ group exhibited some worse clinical characteristics compared to the EFL^gradual^ and EFL^no^ groups (Table 1). Compared to the EFL^gradual^ group, EFL^rapid^ patients had greater airflow obstruction (mean difference (Δ) FEV_1_% predicted = − 11.6%, p = 0.03; Δ FEV_1_/FVC = − 13.3%, p = 0.01), greater hyperinflation (Δ functional residual capacity (FRC) % predicted = 34.9%, p = 0.01), gas trapping (Δ RV % predicted = 29.4%, p = 0.02; Δ RV/total lung capacity (TLC) % predicted = 17.4%, p = 0.049) and greater impact on daily living (Δ SGRQ impact = 13, p = 0.04) (Table 1). The EFL^rapid^ group had worse spirometry measurements, greater hyperinflation and gas trapping, and worse quality of life, driven by activity and impact SGRQ domain scores, compared to the EFL^no^ group (Table 1). Although severity of airflow obstruction was significantly worse for the EFL^rapid^ group, there was considerable overlap of FEV_1_ and FEF_25%–75%_ values between the ELDI groups (FEV_1_ ranges: EFL^rapid^ = 25–69% predicted; EFL^gradual^ = 21–94% predicted; EFL^no^ = 30–87% predicted, Figure S4).

In patients with EFL, ELDI showed a significant correlation with several parameters (Table 2), including FEV_1_% predicted (rho = − 0.52, p < 0.0001), FEV_1_/FVC (rho = − 0.52, p < 0.0001), gas trapping (RV % predicted: rho = 0.37, p < 0.01; RV/TLC: rho = 0.36, p = 0.01), hyperinflation (FRC % predicted: rho = 0.40, p < 0.01) and diffusion capacity of the lungs (DLCO % predicted: rho = − 0.33, p = 0.04; KCO % predicted: rho = − 0.36, p = 0.02). Total SGRQ (rho = 0.39, p = 0.01) together with activity (rho = 0.40, p = 0.01) and impact (rho = 0.37, p = 0.02) domains were positively correlated with ELDI. In patients with EFL, there were no significant associations between lung function measures or questionnaires with either X5_peak-peak_ or ∆X5, apart from the significant associations between lung volumes and X5_peak-peak_ (Table 2; Figure S5).

**Table 2:** Correlations with clinical characteristics in subjects with EFL at baseline (n = 63)

Clinical characteristic	∆X5		X5_peak-peak_		ELDI	
	rho	p	rho	p	rho	p
BMI (kg/m^2^)	0.20	0.11	0.37	0.0032	− 0.23	0.07
Pack years	− 0.32	0.0139	− 0.33	0.0106	− 0.10	0.43
Exacerbations (in the previous 12 months)	− 0.05	0.73	0.08	0.53	− 0.06	0.67
Pre-BD FEV_1_%pred	− 0.12	0.38	0.15	0.26	− 0.52	< 0.0001
Pre-BD FVC %pred	0.01	0.91	0.09	0.51	− 0.17	0.21
Pre-BD FEV_1_/FVC	− 0.12	0.38	0.20	0.15	− 0.52	< 0.0001
Pre-BD FEF_25%-75%_ %pred	− 0.22	0.11	0.01	0.92	− 0.40	0.0024
CAT	0.03	0.80	− 0.01	0.94	0.01	0.97
mMRC	0.03	0.82	0.12	0.35	0.19	0.15
SGRQ total	0.05	0.74	− 0.17	0.31	0.39	0.0137
SGRQ symptoms	− 0.10	0.53	− 0.17	0.29	0.11	0.50
SGRQ activity	0.10	0.55	− 0.16	0.32	0.40	0.0112
SGRQ impact	0.08	0.62	− 0.09	0.59	0.37	0.0184
TLC %pred	− 0.03	0.80	− 0.16	0.23	0.24	0.06
FRC %pred	− 0.02	0.89	− 0.26	0.0435	0.40	0.0018
RV %pred	− 0.06	0.68	− 0.27	0.0381	0.37	0.0039
RV/TLC %pred	− 0.09	0.48	− 0.32	0.0140	0.36	0.0053
DLCO %pred	− 0.09	0.60	0.12	0.46	− 0.33	0.0367
KCO %pred	− 0.15	0.35	0.09	0.57	− 0.36	0.0237
VA %pred	− 0.03	0.87	− 0.08	0.64	0.02	0.90

Missing data: smoking history, n = 4; spirometry, n = 8; CAT & mMRC, n = 4; SGRQ, n = 23; lung volumes, n = 3; DLCO, n = 23

*BD* bronchodilator, *BMI* body mass index, *CAT* COPD assessment test, *DLCO* diffusing capacity for carbon monoxide, *FEV1* forced expiratory volume in 1 s, *FRC* functional residual capacity, *FVC* forced vital capacity, *FEF*_*25%–75%*_ mean forced expiratory flow between 25 and 75% of FVC, *KCO* carbon monoxide transfer coefficient, *mMRC* modified medical research council questionnaire, *RV* residual volume, *SGRQ* St George’s respiratory questionnaire, *TLC* total lung capacity, *VA* alveolar volume

### Repeatability of EFL and ELDI over 6 months

**Fig. 3:**
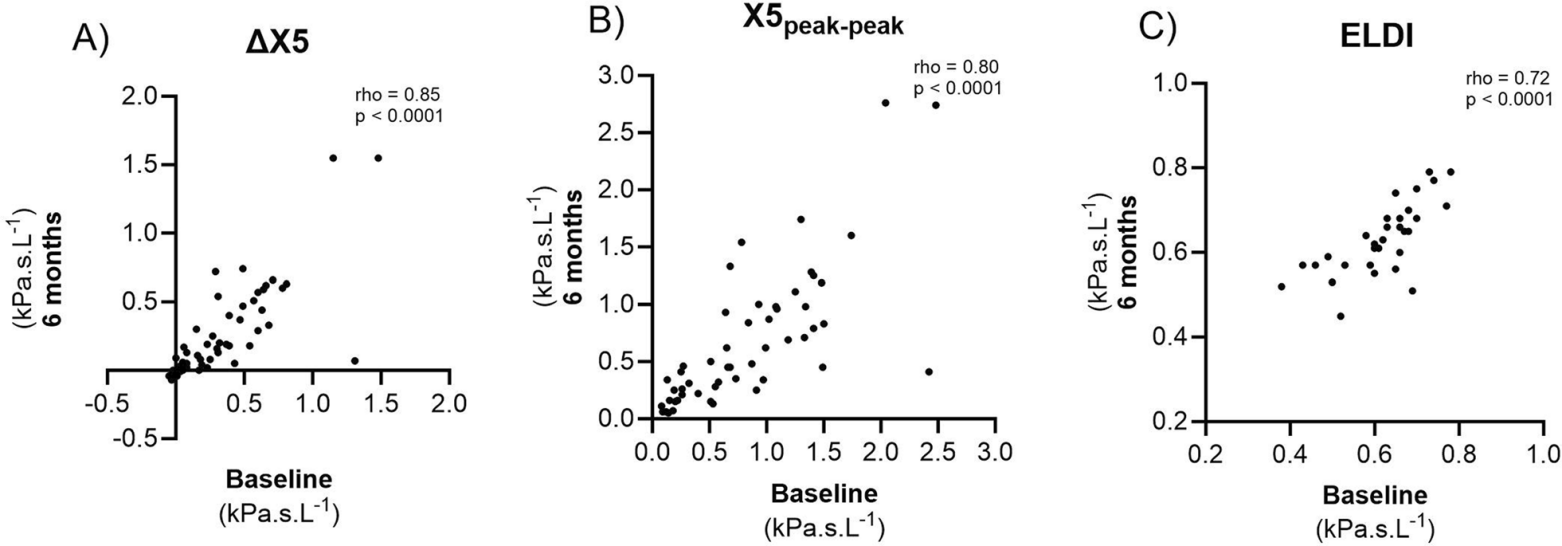
Association between baseline and 6 month measures of EFL: ΔX5 and X5_peak-peak_ in all patients (**A** & **B**, respectively), and ELDI for patients with EFL only (**C**). Patients included in C demonstrated EFLat baseline, defined by a X5_peak-peak_ ≥ 0.59. n = 54 (**A** & **B**) and 31 (**C**). ∆X5: mean difference between inspiratory and expiratory reactance at 5 Hz; X5_peak-peak_: maximum difference between inspiratory and expiratory reactance at 5 Hz; ELDI: EFL Development Index

The association between baseline *versus* 6-month measures, for EFL and ELDI, were investigated using the observational cohort (n = 54; Fig. 3). The magnitude of EFL at baseline and 6 months were positively correlated (ΔX5: rho = 0.85, p < 0.0001; X5_peak-peak_: rho = 0.80, p < 0.0001, Fig. 3A & B respectively), with ICC values of 0.88 and 0.86, respectively. For patients with evidence of EFL at baseline (n = 31), ELDI showed a good correlation at 6 months (rho = 0.72, p < 0.0001, Fig. 3C), with an ICC = 0.80.

All patients with EFL^no^ at baseline remained in the same group 6 months thereafter (Figure S6 A), whilst 74.2% of patients with EFL at baseline also had EFL 6 months thereafter (Figure S6 B&C). The patients who changed EFL status over 6 months (25.8%) had baseline values closer to the threshold for EFL. Changes in clinical characteristics over 6 months were not different between the EFL^rapid^, EFL^gradual^ and EFL^no^ groups (Table S1). Further details are shown in the supplement.

### Treatment effect on ELDI

**Fig. 4:**
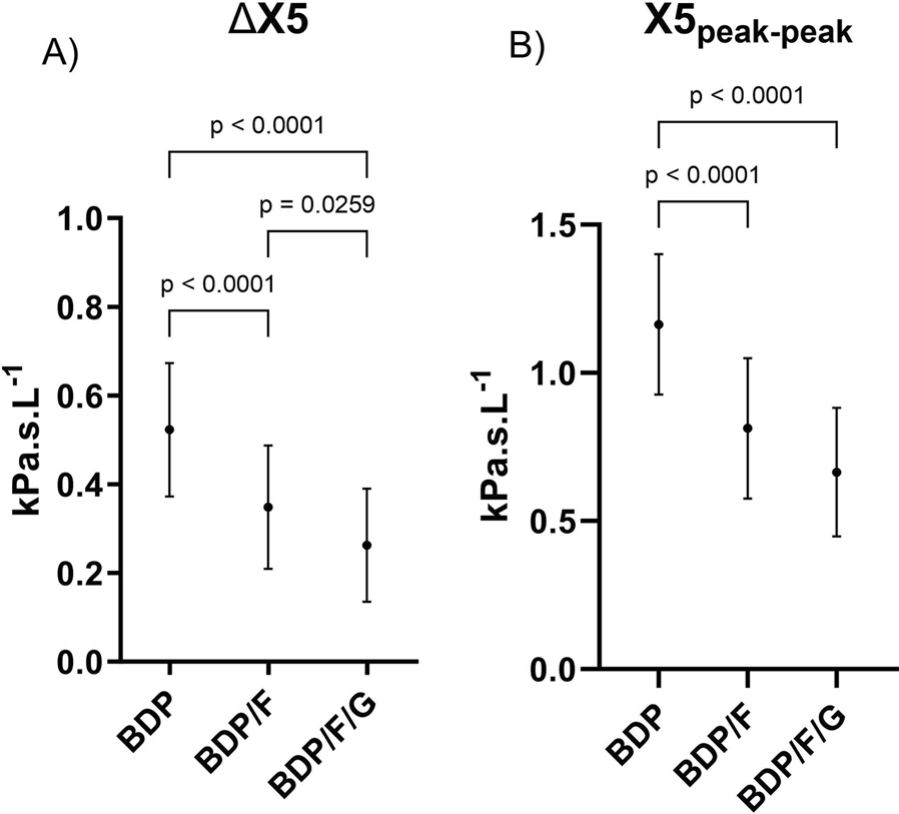
Effect of 5 days treatment with BDP, BDP/F or BDP/F/G on: **A** ∆X5; **B** X5_peak-peak_, in patients with EFL at baseline. Response reported as area under the curve (AUC), calculated as time-weighted AUC for measurements up to 12 h post dose. Data presented as mean (95%CI). ∆X5: mean difference between inspiratory and expiratory reactance at 5 Hz; X5_peak-peak_: maximum difference between inspiratory and expiratory reactance at 5 Hz. BDP: Beclometasone Dipropronate (200 µg twice daily); F: Formoterol (12 µg twice daily); G: Glycopyronium Bromide (20 µg twice daily)

Fifteen of the 22 patients presented with EFL at baseline, after treatment with BDP only. The effect of BDP/F and BDP/F/G treatment in these 15 patients is shown in Fig. 4: BDP/F and BDP/F/G both reduced ∆X5 and X5_peak-peak_, with BDP/F/G causing a significantly greater improvement in ∆X5 compared to BDP/F (p = 0.03, Fig. 4A), while this did not reach significance for X5_peak-peak_, (p = 0.10, Fig. 4B).

**Fig. 5:**
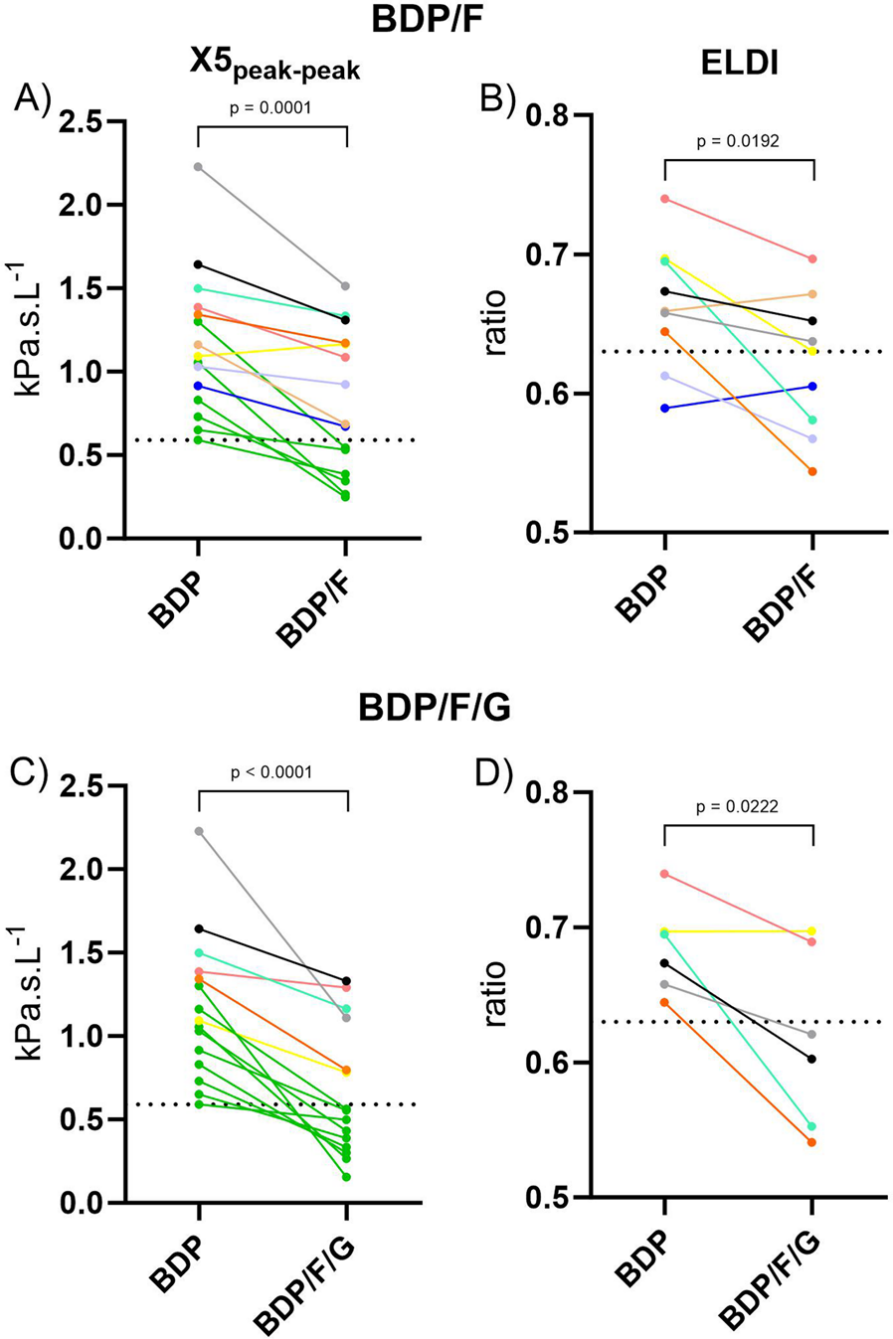
Effect of 5 days treatment with BDP, BDP/F or BDP/F/G on individual change in EFL and ELDI following treatment with BDP/F (**A** & **B**) and BDP/F/G (**C** & **D**). Response reported as area under the curve (AUC), calculated as time-weighted AUC for measurements up to 12 h post final dose on day 5 of treatment. Dotted lines represent thresholds of 0.59 kPa/L/s for EFL (**A** & **C**) and 0.63 for ELDI (**B** & **D**). Data connected by a solid line, represent individual data for patients; for patients in whom EFL was alleviated below the threshold of 0.59 kPa/L/s, data are coloured green (A: n = 6; C: n = 9); for patients in whom EFL persisted (BDP/F: n = 9; BDP/F/G: n = 6), each colour represents a different individual and corresponding ELDI values are plotted for (**A** &**D**). X5_peak-peak_: maximum difference between inspiratory and expiratory reactance at 5 Hz. ELDI: EFL Development Index; BDP: Beclometasone Dipropronate (200 µg twice daily); F: Formoterol (12 µg twice daily); G: Glycopyronium Bromide (20 µg twice daily)

Following treatment with BDP/F and BDP/F/G, 6 and 9 patients no longer met the criteria for EFL (X5_peak-peak_ AUC at day 5 < 0.59 kPa.s.L^−1^), respectively. In these individuals, ELDI at day 5 could not be calculated. Individual changes in X5_peak-peak_ are presented in Fig. 5 (A&C), with representative ELDI values from patients who remained EFL-positive following treatment (Fig. 5 B&D). ELDI was significantly lower following treatment with BDP/F and BDP/F/G (p = 0.02 for both comparisons), with no difference between BDP/F and BDP/F/G (p = 0.71). Improvements in ELDI post-treatment were observed in some patients where the magnitude of EFL was maintained (Fig. 5 B&D, individual raw data examples: figure S7).

## Discussion

In the COPD subgroup with EFL, the novel parameter ELDI has the potential to identify individuals with more rapid onset EFL; ELDI was associated with more severe disease, manifesting as worse airflow obstruction, impaired diffusion capacity of the lungs, greater resting hyperinflation and gas trapping with worse quality of life. Other oscillometry measures of EFL, namely ∆X5 or X5_peak-peak_, were not significantly associated with airflow obstruction, hyperinflation or gas trapping in COPD patients with EFL. These findings implicate the rate of EFL development in the physiological and clinical outcomes in COPD.

Our findings associate small airway collapse with the magnitude of hyperinflation, in agreement with previous studies [7, 8], with our observations now implicating the rate of EFL development with greater gas trapping and hyperinflation. Importantly, amongst COPD patients with EFL, ELDI was not associated with EFL magnitude measured by ∆X5 or X5_peak-peak_. Furthermore, other oscillometry parameters, such as R5-R20 and AX did not distinguish between EFL^rapid^ and EFL^gradual^. These results highlight that ELDI can provide information regarding the dynamics of EFL that cannot be ascertained from other oscillometry parameters. Other key findings were that ELDI exhibited excellent stability over 6 months in an observational cohort setting, while long acting bronchodilator treatment in the TriFLOW study was able to modulate EFL and consequently ELDI.

ELDI was associated with degree of airway limitation, and a significant difference was observed in mean FEV_1_% predicted values between rapid, gradual and no EFL groups. Likewise, there was a difference between the rapid and no EFL groups for FEF^25%−75%^. However, there was a large overlap of FEV_1_ values between all groups (Figure S4), indicating that FEV_1_ values are not a reliable predictor of the likelihood of rapid EFL development on an individual basis. Furthermore, EFL^rapid^ may develop in patients without severe airflow obstruction, as almost a quarter of patients with EFL^rapid^ had FEV_1_ > 50% predicted. This suggests that ELDI identifies a characteristic of small airway dysfunction (and flow limitation) that, on an individual basis, is not consistently associated with FEV_1_. Spirometry parameters are an indirect measure of bronchial obstruction [19, 20]. Other measures of bronchial obstruction such as specific resistance (SRaw) may be more closely related to ELDI, although SRaw measurements were not collected in the present study.

The associations in the observational cohort do not demonstrate causality. However, it is plausible that rapid onset of EFL causes greater gas trapping through airway closure at higher lung volumes; together with increased airway resistance, this contributes to resting hyperinflation, which may worsen upon exercise [21]. There is some evidence that abnormal ∆X5 is associated with exercise intolerance [7, 22] and improves following pulmonary rehabilitation [23], therefore it would be valuable for future studies to investigate whether ELDI further influences exercise capacity or physical activity levels. The pathology responsible for lung volume differences in EFL^rapid^ and EFL^gradual^ groups, in the absence of any difference in other oscillometry parameters, is unclear and may relate to subtle pathological differences. A histology study identified loss of radial alveolar attachments in the small airways as the main pathological feature related to a reduction in FEV_1_ in COPD [24]. Peripheral small airway disease is complex and heterogenous, with altered airway calibre due to airway wall thickening, fibrosis and inflammatory cell infiltration [3]. Additionally, loss of tissue elastic recoil may occur, caused by the disruption of airway-parenchymal interdependence via loss and reduced integrity of alveolar attachments, diaphragm deformation and surfactant deficiency [25]. The nature of EFL may vary between individuals according to the relative contributions of these pathophysiological components. Given that FEV_1_ and FEV_1_/FVC were significantly lower in the EFL^rapid^ group, with an association between impaired gas transfer and ELDI, perhaps the loss of radial alveolar attachments (or at least loss of integrity) through emphysematous destruction leads to airway collapse at higher lung volumes, air trapping and limits capacity for gas transfer. RV/TLC has been proposed as a marker of small airway dysfunction leading to gas trapping [26–28], and our results indicate faster development of EFL in those with greater small airway disease.

EFL^gradual^ patients demonstrated differences to EFL^no^, including greater airflow obstruction and more gas trapping. However, symptoms scores and degree of hyperinflation were comparable between groups. These findings may signify resting hyperinflation with more rapid EFL development, although whether this is the cause or a consequence cannot be determined from the associations in the cohort analysis. A higher body mass index (BMI) was observed for EFL^gradual^ patients compared to EFL^no^ (see supplement). An association between BMI and/or obesity and EFL has been demonstrated previously [29], therefore the EFL^gradual^ group may constitute a mixture of patients with EFL induced by mechanical alterations associated with obesity [30] and those with an intermediate state of EFL attributed to small airway disease.

The TriFLOW analysis demonstrated that EFL can be improved by long acting bronchodilator therapy, with ELDI shifting towards less rapid development. In some individuals, EFL^gradual^ may represent an intermediate state of EFL, between EFL^rapid^ and EFL^no^, which can be modified by bronchodilator treatment. EFL was improved upon by treatment with either ultrafine BDP/F or BDP/F/G. In patients with EFL which persisted following treatment, ELDI improved significantly with BDP/F and BDP/F/G, albeit in a small sample size. Whilst both the magnitude of EFL (∆X5 or X5_peak-peak_) and nature of EFL development (ELDI) improved following treatment, these measures did not demonstrate an association with one another, and ELDI was associated with worse baseline clinical characteristics in patients with EFL. These findings highlight the importance of considering the nature of EFL development in addition to the magnitude. Collectively, changes in EFL and ELDI following treatment, together with other lung function changes reported from the TriFLOW study elsewhere [11], suggest that lung volume dependant choke points may move closer to residual volume (RV) after bronchodilators, reducing gas trapping and allowing greater volume to be exhaled during forced spirometry. It is unclear if this is a function of magnitude and/or rate of EFL development, although we suggest that the interpretation of EFL requires consideration of both.

Previous studies have demonstrated that high levels of EFL are relatively stable over time [5, 8]. Our data now shows that ELDI was consistent in those with EFL. Most patients did not change their categorisation over 6 months, however those with baseline values closer to the ELDI threshold showed some variability, in keeping with natural variation across a binary threshold. This threshold was determined using the mean cohort value; further investigation is required to identify a threshold for clinical practice. Changes in clinical characteristics between visits were similar between all groups, supporting no change in clinical status despite some shifts in ELDI categorisation.

While this paper focuses on ELDI, there are potentially other possible ways to analyse the shape of X5 loops and the development of EFL (illustrated in Figure S8). The rate (i.e. gradient) at which X5 decreases during early exhalation could be calculated, however this requires a more complex calculation of exported raw data which may not be available with all commercial equipment. Furthermore, this would not account for different times of EFL onset (earlier versus later during exhalation). The percent of tidal volume at which EFL begins could be evaluated, but this also requires data export, and does not consider the overall magnitude of EFL. The advantages of ELDI are that it considers EFL magnitude, gives an indication of speed of EFL development, whilst also being a simple calculation that can be made from historical data.

We have demonstrated that rapid development of EFL was associated with more severe disease and worse gas trapping with hyperinflation, when compared to COPD patients with gradual EFL. In patients with EFL, the speed at which EFL developed during exhalation (i.e. ELDI) was associated with worse clinical characteristics, while the magnitude of EFL (i.e. ∆X_5_ or X5_peak-peak_) was not. Furthermore, rapid development of EFL was a relatively stable phenomenon and may be sensitive to changes in response to inhaled therapy in COPD. Overall, we propose ELDI as a clinically useful COPD marker associated with clinical and physiological characteristics.

## Supplementary Information

Additional file 1.

## Data Availability

The datasets generated and/or analysed during the current study and additional related documents are not publicly available.

## Abbreviations

COPD: Chronic obstructive pulmonary disease
EFL: Expiratory flow limitation
X5_in,mean_: Mean inspiratory reactance
X5_ex,mean_: Mean expiratory reactance
kPa: Kilopascal
s: Second(s)
L: Litres
∆X5: Difference between mean inspiratory and mean expiratory reactance
X5_peak-peak_: Difference between max inspiratory and minimum expiratory reactance
EFL^rapid^: Rapid onset of expiratory flow limitation
EFL^gradual^: Gradual onset of expiratory flow limitation
X5_ex,min_: Minimum expiratory reactance
ELDI: Expiratory flow limitation development index
NHS: National healthy service
UK: United kingdom
GOLD: Global initiative for chronic obstructive pulmonary disease
FEV_1_: Forced expiratory volume in 1 s
FVC: Forced vital capacity
IOS: Impulse oscillometry
DLCO: Diffusing capacity of the lungs for carbon monoxide
BDP: Beclomethasone dipropionate
F: Formoterol
G: Glycopyrronium
ATS: American Thoracic Society
ERS: European Respiratory Society
EFL^no^: No expiratory flow limitation
ANOVA: Analysis of variance
ICC: Intraclass correlation coefficient
R5-R20: Respiratory resistance at 5 Hz minus respiratory resistance at 20 Hz, a.k.a. small airway resistance
AX: Reactance area, a.k.a. peripheral airway distensibility
FRC: Functional residual capacity
RV: Residual volume
TLC: Total lung capacity
SGRQ: St Georges respiratory questionnaire
rho: Correlation coefficient
KCO: Carbon monoxide transfer coefficient
BMI: Body mass index
