# Supplementary material for: Expiratory flow limitation development index (ELDI): a novel method of assessing respiratory mechanics in COPD
Source: Respir Res. 2024 Oct 3;25:357. doi: 10.1186/s12931-024-02972-2 (PMC11448286; doi:10.1186/s12931-024-02972-2)
Supplement: Supplementary file 1 — Additional file 1. [file 12931_2024_2972_MOESM1_ESM.docx]

**Supplementary material**

**Expiratory flow limitation development index (ELDI): A novel method of assessing respiratory mechanics in COPD.**

James Dean^1,2^, Stephen J Fowler^1^, Dave Singh^1,2^ and Augusta Beech^1,2^

^1^ Division of Infection, Immunity and Respiratory Medicine, School of Biological Sciences, Faculty of Biology, Medicine and Health, Manchester Academic Health Science Centre, The University of Manchester, Manchester University NHS Foundation Trust, Manchester, M23 9LT, United Kingdom.

^2^ Medicines Evaluation Unit, Manchester, M23 9QZ, United Kingdom, UK.

Correspondence: James Dean

Medicines Evaluation Unit, Southmoor Road, Manchester, M23 9QZ, UK

Email: jdean@meu.org.uk

**Methods**

*Observational cohort*

The observational cohort^1^ comprised of highly symptomatic patients with a modified Medical Research Council (mMRC)^2^ score ≥2 and a chronic obstructive pulmonary disease (COPD) Assessment Test (CAT) score ≥15^3^.

*TriFLOW study*

The TriFLOW study comprised of patients with evidence of gas trapping identified by a Residual Volume (RV) >120% predicted^4^. Patients who completed screening but did not meet the study inclusion criteria (n = 23) were included in the overall cohort. Treatment effect was assessed from patients who presented with expiratory flow limitation (EFL) at baseline and completed all study treatments (n = 15/22). Eligible patients commenced a 10–28 day run-in period receiving beclomethasone dipropionate (BDP, 100 µg pressurised metered-dose inhaler [pMDI], 2 puffs, twice daily) in place of current inhaled corticosteroid (ICS) treatment. Long-acting bronchodilator treatments were withdrawn and replaced with short acting bronchodilators for use, as needed. After run-in, a baseline visit was completed, followed by two treatment periods of 5 days each, separated by a washout period of 7–21 days during which BDP and short acting bronchodilators were used.

Patients were randomised (with a 1:1 ratio), to receive triple therapy (Trimbow: BDP/Formoterol(F)/Glycopryrronium(G), 100/6/10 µg pMDI, 2 puffs, twice daily) followed by dual therapy (Fostair: BDP/F, 100/6 µg pMDI, 2 puffs, twice daily) or vice-versa. BDP/F/G or BDP/F treatment was commenced on the morning of day 1 and a final dose given on the morning of day 5 (total of 9 doses). At the baseline visit, and on day 5 of each treatment period, impulse oscillometry (IOS), spirometry, and whole body plethysmography were performed (in that order) prior to the morning dose. IOS and spirometry were then repeated 30 min and 1, 2, 4, 6, 8, 10 and 12 h post-dose. Plethysmography was repeated at 1, 2, 4, 8 and 12 h post-dose. Time weighted area under the curve (AUC) was calculated for each subject and used for analysis On day 1 of each treatment period, patients performed spirometry, IOS, and whole body plethysmography prior to the first administration of BDP/F/G or BDP/F.

*Symptom Questionnaires*

For the observational cohort, the mMRC, CAT, and St George’s Respiratory Questionnaire (SGRQ)^5^ was administered at baseline and 6 months. For the TriFLOW study, the mMRC and CAT questionnaires were administered at screening visit.

*Lung function*

For the observational cohort and screening visits for the TriFLOW study, short acting bronchodilators were withheld for 6 hours, long-acting bronchodilators, anticholinergics, theophyllines and leukotriene receptor antagonists were withheld for up to 24 hours prior to lung function testing. Post-bronchodilator spirometry was performed to confirm GOLD definition of airflow obstruction, but was not reported in the analysis.

*Oscillometry*

The IOS method was employed for oscillometry measurements (MasterScreen; Erich Jaeger, Hoechbery, DEK), and equipment verification was performed prior to use using a resistive load of 0.20 kPa/L/s, where readings were required to be within ±0.01 kPa/L/s. IOS measurements were recorded prior to other lung function measurements. The patient, sitting with an upright posture and a slight elevation of the chin, performed tidal breathing through a mouthpiece with tongue depressor, whilst wearing a nose clip and supporting their cheeks with their hands. A sound wave was generated by a loudspeaker applied every 0.2 seconds over a 30 second timeframe. A minimum of 3 technically acceptable results were recorded and mean values reported, technically acceptable attempts should be devoid of artefacts (in pressure, flow and volume), have a coherence ≥0.7 at 5 and 20 Hz, and be repeatable between measurements. Repeatable measurements were defined by resistance at 5 Hz (R5), resistance at 20 Hz (R20) and resonant frequency (Fres) values within 10% of the mean of three values.

Inspiratory and expiratory data were averaged over multiple tidal breaths, which has been validated against the breath-by-breath method where differences between inspiratory reactance at 5 Hz (X5_in_) and expiratory reactance at 5 Hz (X5_ex_) are calculated per breath and then averaged^6^.

*Lung Volumes*

When assessing lung volumes, hyperinflation was calculated as functional residual capacity (FRC) % predicted, and gas trapping as residual volume/total lung capacity (RV/TLC) % predicted.

**Results**

*Repeatability of EFL and ELDI over 6 months*

The majority of subjects returned for a repeat visit after 6 months. Subjects who did not return were either uncontactable (n = 10), unable to produce acceptable oscillometry results (n = 1) or were withdrawn due to change in medical circumstances (n = 4) ^1^.

For patients that moved from EFL to EFL^no^ groups (n = 8), baseline EFL values were closer to the threshold (for X5_peak-peak_) compared to those in which EFL persisted (n = 23), although this difference was not statistically significant (0.89 versus 1.25 kPa/s/L^-1^, p = 0.22). For patients in which EFL persisted, but ELDI status changed (EFL^grad^ > EFL^rapid^, n = 1; EFL^rapid^ > EFL^grad^, n = 2), values were closer to the threshold (for ELDI) compared to their stable counterparts, however this was not statistically significant.

**Discussion**

Those with gradual development of EFL (EFL^gradual^) were found to have significantly higher body mass index (BMI) values than those without EFL (EFL^no^), which was not the case for those with rapid EFL (EFL^rapid^). It is unclear whether our results indicate an increased stiffness of the respiratory system in obesity preventing sudden onset of EFL, or an inconsequential difference between our groups. Fat deposition in the thorax and abdomen can result in a restrictive ventilatory defect, which presents as reduced lung volumes, reduced elastic recoil, and EFL^7^. Consequently, COPD and obesity have opposite effects on FRC and RV, the former hyperinflating their volume, and the latter decreasing them. Patients in our cohort with obesity may have had milder EFL that was exacerbated by reduced lung volumes, resulting in tidal breathing occurring closer to the choke points (i.e. near RV). The relationship between BMI, lung volumes, and EFL requires further investigation.

**Table S1:** Change in clinical characteristics over 6 months between rapid, gradual and no EFL groups.

| **Clinical characteristic** | **Absolute change over 6 months (ALL, n = 54)** | **Absolute change over 6 months**  **(EFL only, n=31)** | **EFL^rapid^**  **(n = 15)** | **EFL^gradual^**  **(n = 16)** | **p-value** | **EFL^no^**  **(n = 23)** | **p-value**  **vs EFL^rapid^** | **p**  **-value**  **vs EFL^gradual^** |
| --- | --- | --- | --- | --- | --- | --- | --- | --- |
| BMI (kg/m^2^) | -0.3 [-5.3 – 2.7] | -0.3 [-2.9 – 2.7] | -0.3 [-1.4 – 2.7] | -0.2 [-2.9 – 0.9] | >0.99 | -0.3 [-5.3 – 1.0] | >0.99 | >0.99 |
| Pre-BD FEV_1_ % pred | -0.9 (6.2) | -0.0 (0.14) | 0.1 (6.2) | -2.3 (4.2) | 0.52 | -0.9 (7.3) | 0.89 | 0.75 |
| Pre-BD FVC % pred | 9.5 (11.0) | -1.2 (5.3) | 8.9 (15.0) | 9.1 (10.4) | 0.99 | 9.6 (8.8) | 0.98 | 0.99 |
| Pre-BD FEV_1_/FVC (%) | -1.2 (3.8) | -0.8 (3.3) | 0.3 (3.3) | -1.8 (2.9) | 0.30 | -1.7 (4.6) | 0.29 | 0.99 |
| Pre-BD FEF_25%-75%_ % pred | -1.3 (6.5) | -0.5 (5.2) | 1.8 (3.6) | -3.0 (5.6) | 0.11 | -2.1 (8.0) | 0.18 | 0.90 |
| CAT | -0.8 (5.8) | -0.7 (5.1) | -1.8 (5.5) | 0.4 (4.7) | 0.56 | -1.2 (6.8) | 0.95 | 0.68 |
| mMRC | 0 [-4 – 1] | 0.0 [-3.0 – 1.0] | 0.0 [-3.0 – 1.0] | 0.0 [-3.0 – 0.0] | >0.99 | 0.0 [-3.0 – 1.0] | >0.99 | >0.99 |
| SGRQ Symptom | -4.4 (14.9) | -5.1 (14.9) | -0.7 (16.2) | -9.2 (12.7) | 0.26 | -3.1 (15.2) | 0.88 | 0.42 |
| SGRQ Activity | -3.3 (13.3) | -2.7 (14.0) | -2.8 (14.2) | -2.6 (14.3) | 0.99 | -4.1 (12.5) | 0.95 | 0.94 |
| SGRQ Impact | -1.4 (12.4) | -1.2 (10.9) | -1.8 (14.5) | -0.7 (6.5) | 0.97 | -1.6 (14.4) | 0.99 | 0.97 |
| SGRQ Total | -2.5 (9.6) | -2.3 (8.9) | -2.0 (12.1) | -2.7 (4.8) | 0.98 | -2.7 (10.6) | 0.98 | >0.99 |
| TLC % pred | 1.2 (6.0) | 0.7 (6.1) | 2.2 (6.5) | -0.8 (5.5) | 0.41 | 1.2 (6.5) | 0.88 | 0.63 |
| FRC % pred | 2.5 (12.6) | 0.8 (11.7) | -0.3 (12.6) | 1.8 (11.2) | 0.91 | 5.2 (16.2) | 0.50 | 0.75 |
| RV % pred | 0.8 [-49.7 – 35.4] | -1.0 [-92.4 – 35.4] | 2.6 [-92.4 – 35.4] | -3.2 [-15.6 – 20.1] | >0.99 | 4.4 [-49.7 – 68.2] | >0.99 | >0.99 |
| RV/TLC (%) | -1.3 [-33.9 – 31.5] | -1.5 [-61.8 – 31.2] | -1.0 [-61.8 – 22.1] | -2.6 [-13.1 – 31.5] | >0.99 | -0.8 [-33.9 – 15.4] | >0.99 | >0.99 |
| DLCO % pred | -1.0 [-28.0 – 26.0] | 0.0 [-26.0 – 9.6] | 1.3 [-26.0 – 9.6] | -1.4 [-14.4 – 5.7] | >0.99 | -4.0 [-28.0 – 26.0] | 0.27 | >0.99 |
| KCO % pred | -1.6 [-34.0 – 20.0] | -1.0 [-34.0 – 10.8] | 0.0 [-34.0 – 10.8] | -2.2 [-17.0 – 10.0] | >0.99 | -5.0 [-30.0 – 20.0] | 0.65 | >0.99 |
| VA % pred | 0.0 (6.6) | 1.1 (6.3) | 1.8 (5.2) | 0.4 (7.4) | 0.83 | 0.4 (6.9) | 0.80 | 0.99 |

Note. Not all data available for each subject. Missing data: Pre-BD FEV_1_ % pred, FVC % pred and FEV_1_/FVC – n = 1; Pre-BD FEF_25%-75%_ % pred – n = 4; TLC % pred and FRC % pred – n = 3; RV % pred and RV/TLC (%) – n = 4; DLCO % pred, KCO % pred and VA % pred – n = 1.

Abbreviations: BD, bronchodilator; BMI, body mass index; CAT, COPD assessment test; DLCO, diffusing capacity for carbon monoxide; FEV1, forced expiratory volume in 1 second; FRC, functional residual capacity; FVC, forced vital capacity; FEF_25%-75%_, mean forced expiratory flow between 25% and 75% of FVC; KCO, carbon monoxide transfer coefficient; mMRC, modified medical research council questionnaire; RV, residual volume; SGRQ, St George’s respiratory questionnaire; TLC, total lung capacity; VA, alveolar volume.

**Supplemental Figures**


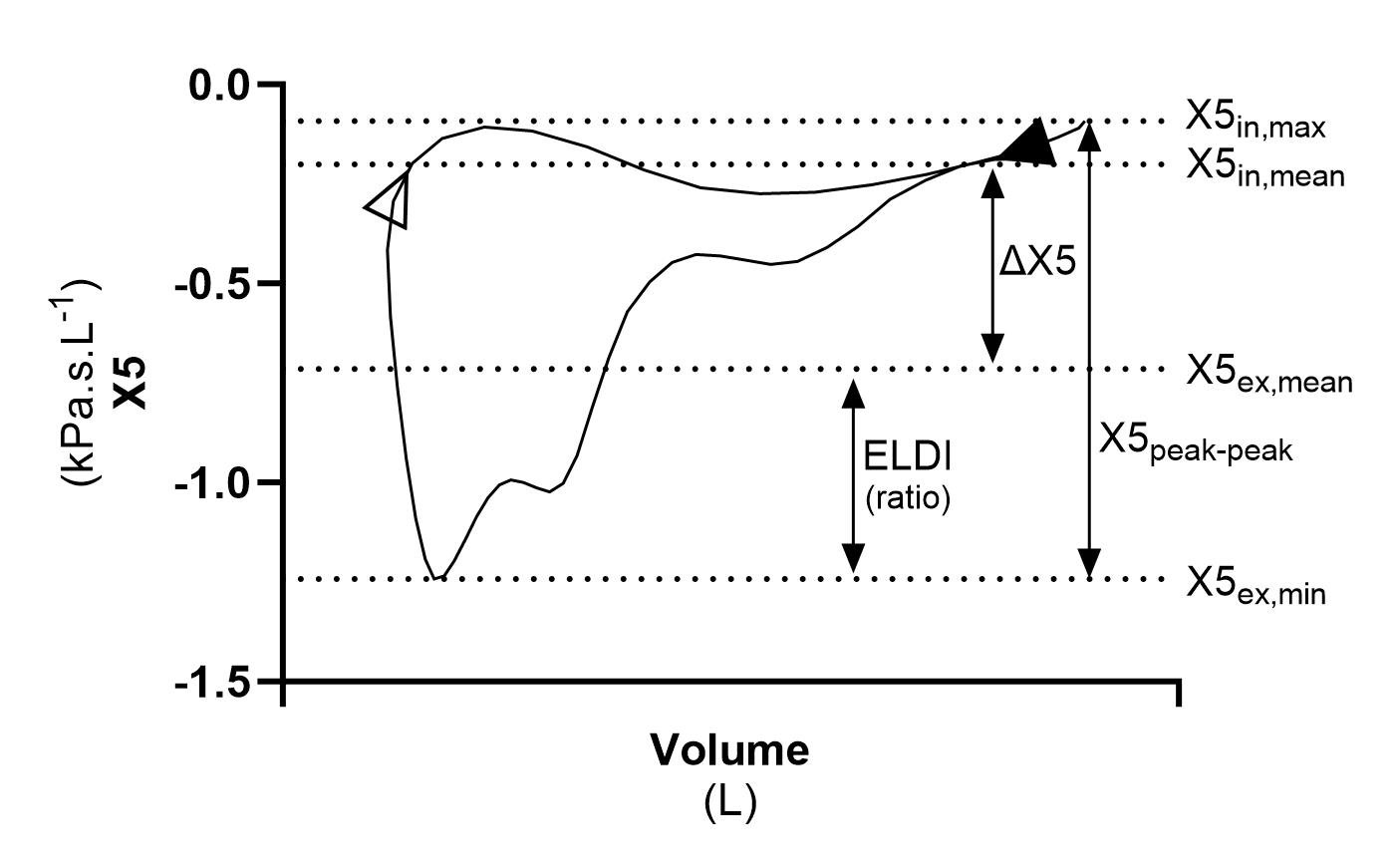


**Figure S1:** Reactance-volume loop for a COPD patient, using reactance at 5 Hz (X5), including EFL Development Index (ELDI = X5_ex,mean_/_min_). EFL - ∆X5 (mean difference ≥0.28 between inspiration and expiration); X5_peak-peak_ (maximum difference ≥0.59 between inspiration and expiration). The more rapid the onset of EFL, the closer X5_ex,mean_ is to X5_ex,min_, resulting in a higher ELDI ratio. ELDI is not calculated for subjects without EFL. X5 – reactance at 5 Hertz; X5_in,mean_ – mean X5 during inspiration; X5_ex,mean_ – mean X5 during expiration; X5_in,max_ – maximum X5 during inspiration; X5_ex,min_ – minimum X5 during expiration. ▲– start of expiration; ∆ - start of inspiration.


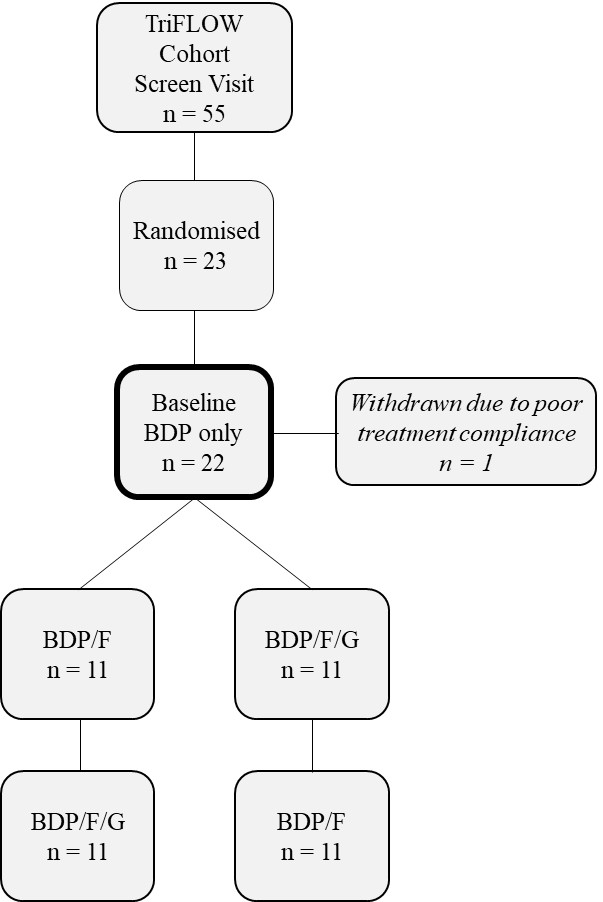


**Figure S2:** Flow diagram of COPD patients selected from the TriFlow study. Baseline visit was performed after 10-28 days of BDP only dosing. Treatment visits were performed after 5 days BID dosing with either BDP/F or BDP/F/G, in a crossover design, with a washout of 7-21 days. BID: twice daily dosing; BDP: Beclometasone Dipropronate (200µg twice daily); F: Formoterol (12µg twice daily); G: Glycopyronium Bromide (20µg twice daily).


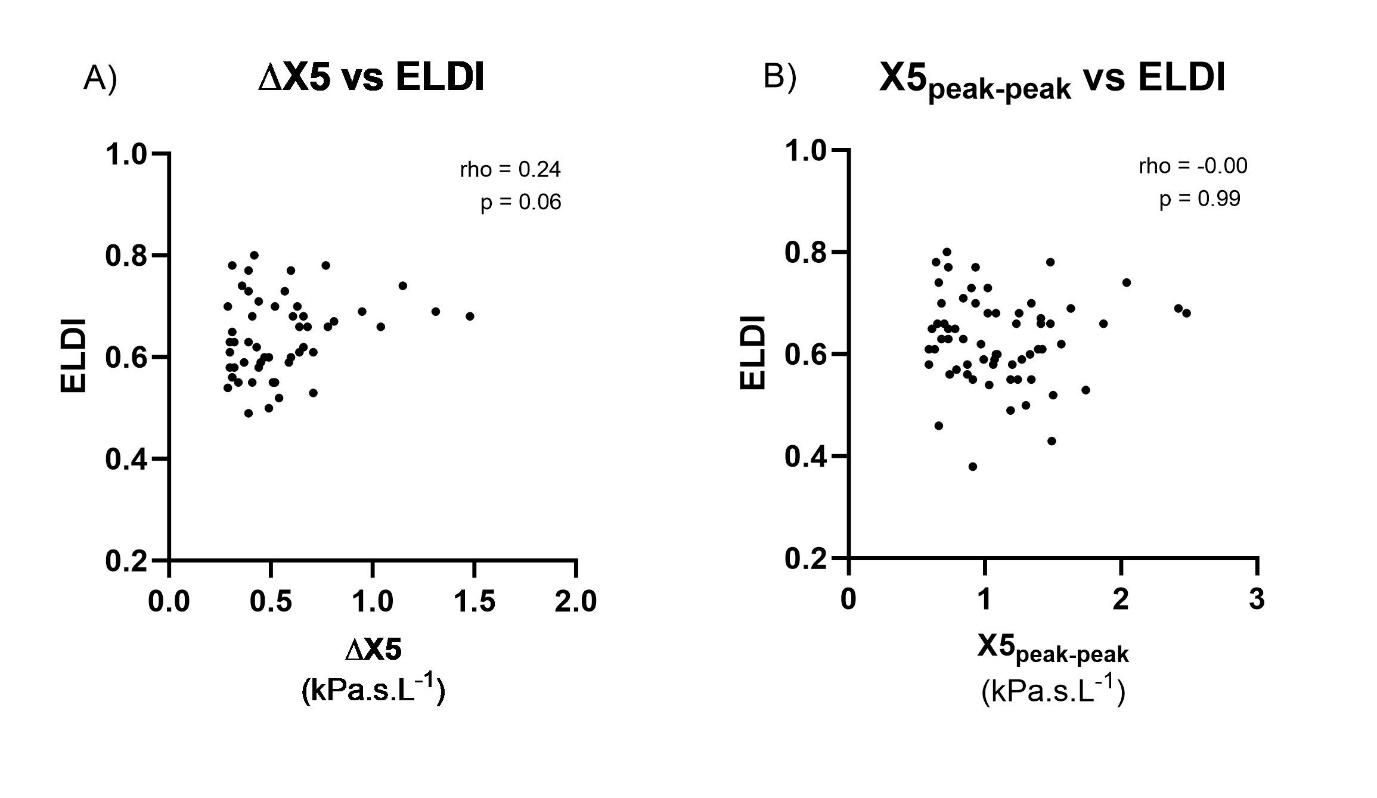


**Figure S3:** Correlation in patients with EFL between ELDI and A) ∆X5; B) X5_peak-peak_. ∆X5: mean difference between inspiratory and expiratory reactance at 5 hertz; ELDI: EFL Development Index; X5_peak-peak_: maximum difference between inspiratory and expiratory reactance at 5 hertz.

**Figure S4:** Scatterplot of A) FEV_1_ and B) FEF_25%-75%_ % predicted for EFL^rapid^, EFL^gradual^ and EFL^no^. Lines and error bars represent mean (SD); FEV_1_: forced expiratory volume in 1 second. EFL: expiratory flow limitation; FEF_25%-75%_: forced expiratory flow between 25% and 75% of FVC.


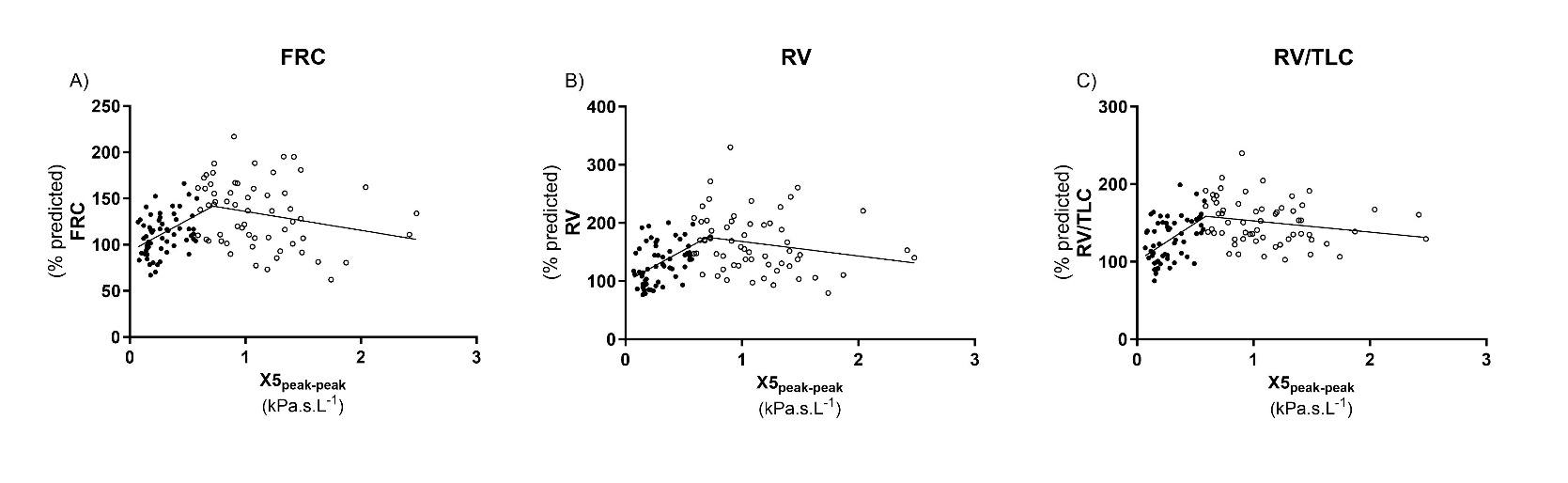


Figure S5: Scatter plots in all patients (EFL^rapid^, EFL^gradual^ and EFL^no^) between X5_peak-peak_ and A) FRC; B) RV; C) RV/TLC. ●: No EFL; ○: EFL. Segmental linear regression lines showing a positive association in none EFL patients, and a negative association in EFL patients. X5_peak-peak_: maximum difference between inspiratory and expiratory reactance at 5 hertz; FRC: functional residual capacity; RV: residual volume; TLC: total lung capacity.


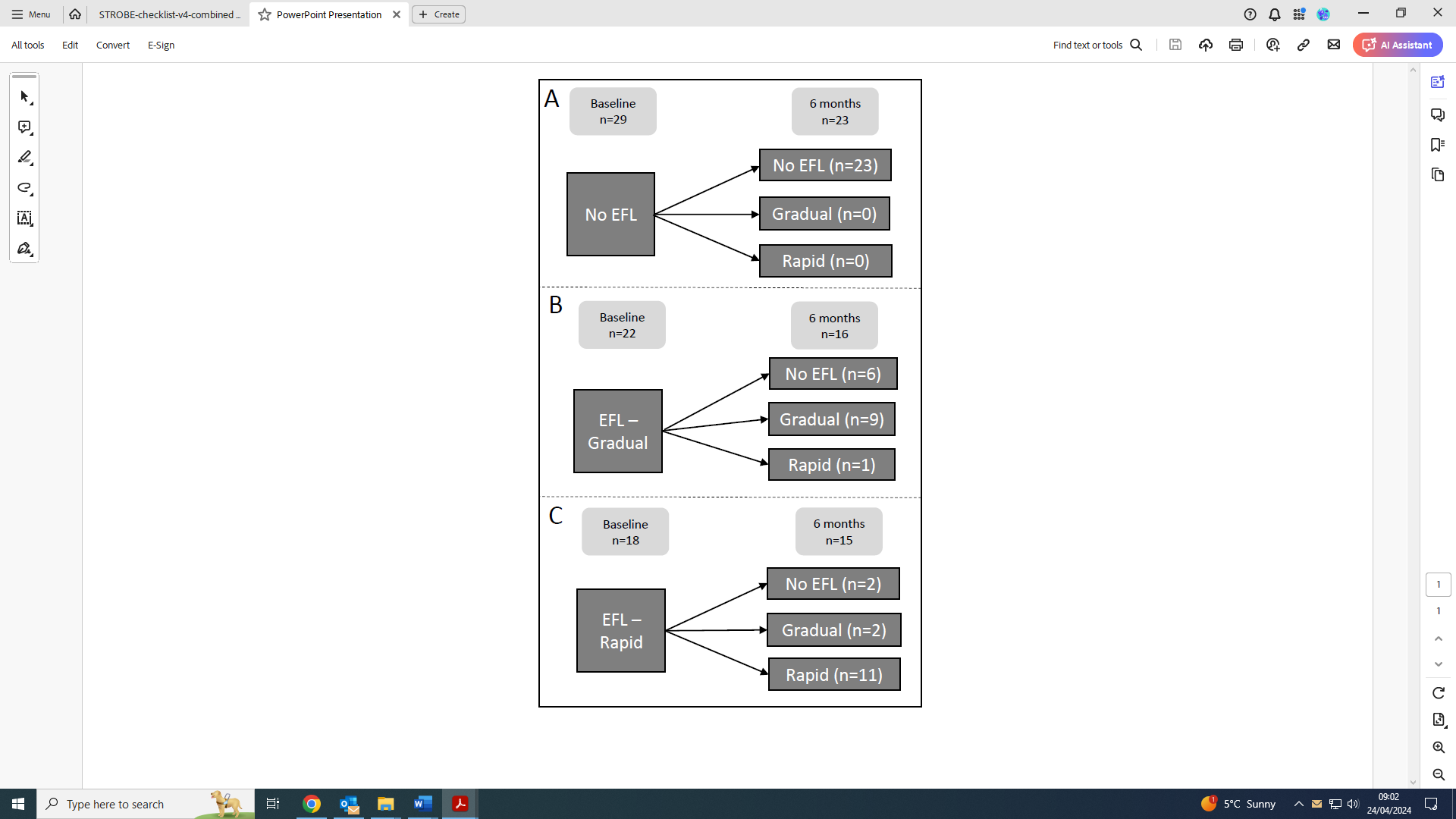


**Figure S6:** Change in EFL status over 6 months in the repeatability cohort. EFL defined as X5_peak-peak_ ≥0.59. Baseline n=69; 6 months n=54. A) Patients with no EFL at baseline; B) Patients with gradual EFL at baseline; C) Patients with rapid EFL at baseline. EFL – expiratory flow limitation.


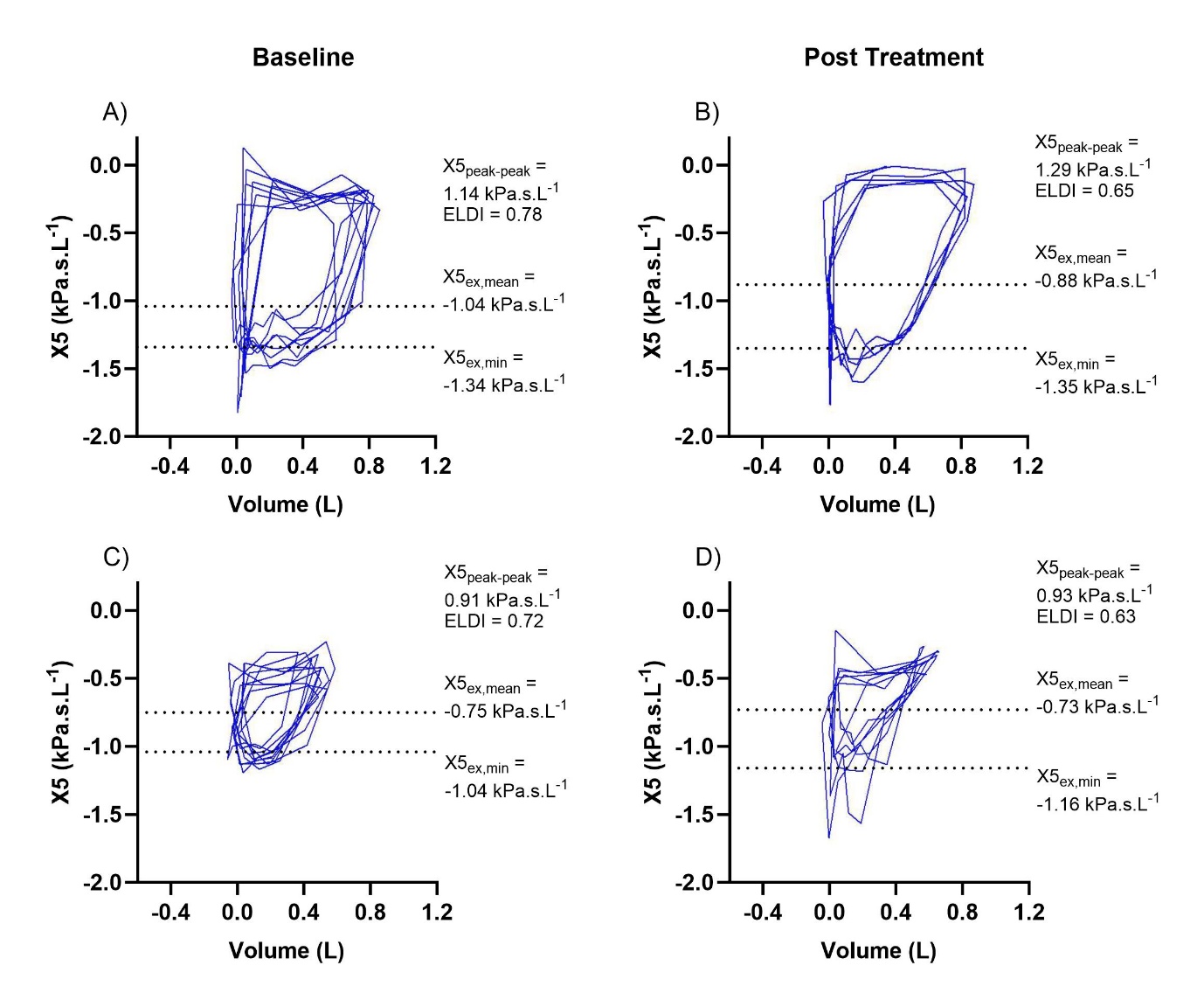


**Figure S7:** Reactance-volume loops of two patients who experienced an improvement in ELDI but not X5_peak-peak_ following treatment. Improvement in ELDI can be visualised by closing of the reactance-volume loop, becoming more triangular. Patient 1: A – trial taken from baseline visit (i.e. post BDP only); B - trial taken from post BDP/F visit. Patient 2: C – trial taken from baseline visit (i.e. post BDP only); D - trial taken from post BDP/F visit. X5: reactance at 5 hertz; X5_peak-peak_: maximum difference between inspiratory and expiratory reactance at 5 hertz; X5_ex,mean_: mean expiratory reactance at 5 hertz; X5_ex,min_: minimum expiratory reactance at 5 hertz; ELDI: EFL development index.


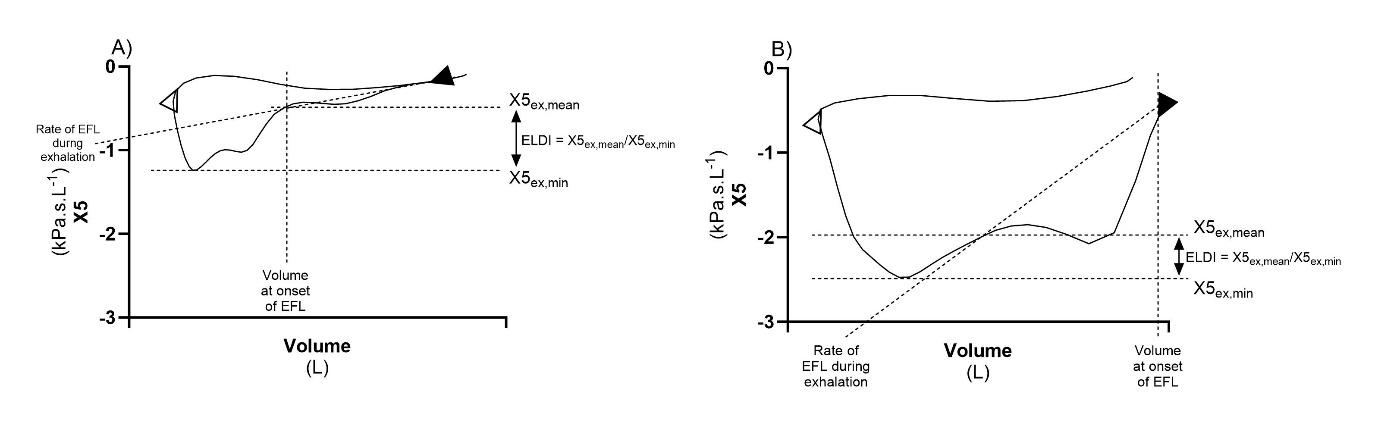


**Figure S8**: Reactance-volume loops of two example patients who: A) experience EFL late during exhalation; B) experience EFL at onset of exhalation. Potential methods of quantifying EFL development shown: ELDI = X5_ex,mean_/X5_ex,min_; Volume at onset of EFL = Volume at which X5 suddenly decreases during exhalation; Rate of EFL during exhalation = average rate at which X5 decreases during exhalation. EFL: expiratory flow limitation; X5: reactance at 5 hertz; X5_ex,mean_: mean expiratory reactance at 5 hertz; X5_ex,min_: minimum expiratory reactance at 5 hertz; ELDI: EFL development index.

**References**

1. Beech A, Jackson N, Dean J, et al. Expiratory flow limitation in a cohort of highly symptomatic COPD patients. ERJ Open Res 2022; 9(6).
2. Mahler DA, Wells CK. Evaluation of clinical methods for rating dyspnea. Chest 1988; 93(3): 580-586.
3. Jones PW, Harding G, Berry P, et al. Development and first validation of the COPD Assessment Test. Eur Respir J 2009; 34: 648-654.
4. Dean J, Panainte C, Khan N, et al. The TRIFLOW study: a randomised, cross-over study evaluating the effects of extrafine beclometason/formoterol/glycopyrronium on gas trapping in COPD. Respir Res 2020; 21(323).
5. Jones PW, Quirk FH, Baveystock CM. The St. George’s Respiratory Questionnaire. Reso Med 1991; 85(suppl B): 2531.
6. Aarli BB, Calverley PM, Jensen RL, et al. Variability of within-breath reactance in COPD patients and its association with dyspnoea. Eur Respir J 2015; 45: 625–634.
7. de Albuquerque CG, de Andrade FMD, de Almeida Rocha MA, et al. Determining respiratory system resistance and reactance by impulse oscillometry in obese individuals. J Bras Pneumol 2015; 41(5): 422-428.
